# Social media usage and health promoting lifestyle in profile related socio-demographic factors in Turkey

**DOI:** 10.15171/hpp.2020.13

**Published:** 2020-01-28

**Authors:** Dilek Güleç, Selen Yılmaz Işıkhan, Emine Orhaner

**Affiliations:** ^1^Polatlı Vocational School of Health Services, Hacettepe University, Sıhhıye, 06100, Ankara, Turkey; ^2^Vocational School of Social Sciences, Hacettepe University, Opera, 06100, Ankara, Turkey; ^3^Faculty of Economics and Administrative Sciences, Hacı Bayram University, Beşevler, 06500, Ankara, Turkey

**Keywords:** Social media, Healthy lifestyle, Health promotion, Multivariate analysis

## Abstract

**Background:** Social media represents a revolutionary new trend that offers opportunities for and threats toward modifying health behaviours. Although social media has considerable health promotion and education tools, this article summarizes the relationship between the health promoting lifestyle and Facebook usage, as Facebook is one of the most popular tools in social media.

**Methods:** We carried out a cross-sectional, descriptive study with 423 Facebook users living in Ankara, Turkey. Nonlinear canonical correlation analysis (OVERALS) was used to describe the complex links between health behaviours, social media usage and demographic characteristics.

**Results:** In this study, a two-dimensional solution with an actual fit measure equal to 0.958was found, and this solution can be interpreted as about 48% of the explained variance. This two-dimensional result shows the relationships and differences between healthy lifestyle subdimensions, social media usage and some demographic characteristics.

**Conclusion:** Using OVERALS, we found evidences supporting associations among social media use, health promoting lifestyle and socio-demographic factors. Further, the complex correlations among these variables were interpreted.

## Introduction

Social relationships have been linked to positive health behaviours, good mental and physical health, and lower mortality risk. Adults who are more socially connected are healthier and live longer than their more isolated peers.^1-3^ Therewithal, emotional support provided by social ties, such as being married, having children and religious involvement enhances positive health behaviours and reduces the risk of unhealthy behaviours.^4-7^

An increasing fraction of today’s social interactions occur using online social media as communication channels.^8^ Social media users spend an average of 118 minutes worldwide and 181 minutes in Turkey using social media each day.^9,10^ In Turkey 92% of internet users use Facebook.^10^

Researchers are interested in social media and health issues because they have become an important part of our everyday lives. Possible impacts of Facebook use on health, education, social life, consumption etc. reported in the previous literature. Studies on the health implications of Facebook use have generated many important results. Facebook use has been shown to be associated with many mental health domains such as Facebook addiction, psychological distress (i.e., depression, anxiety, etc.) and well-being (life satisfaction, positive mental health) anxiety, body image and disordered eating, drinking cognitions and alcohol use.^11,12^ For all that, the heightened interpersonal connectivity afforded by social media was associated with an overall increase in psychological well-being. Ellison et al^13^ reported that intensive use of Facebook predicts social capital accumulation and is found to interact with measures of psychological well-being. In addition, some studies show that engagement with Facebook and number of Facebook friends has a positive association with subjective well-being,^14^ which predicts an array of mental and physical health consequences.^11,15^

Facebook usage has been associated with many health outcomes, but little is known about the relationships between Facebook use and health-promoting behaviors. This article largely focuses on exploring the relationships between social media and the health-promoting lifestyle. We use Facebook as a research tool in order to determine the relationship. As of January 2017, Facebook, registered as having 1.871 billion active users all over the world, was the most popular online social networking application. This is also the case in Turkey, where it has 48 million active users.^16^ As a result, Facebook was determined to be a viable research tool both for its widespread use and its features that provide an invaluable resource for fulfilling the basic human need for social connection. It is hoped that the results of this study will provide information to policy makers for developing health promotion strategies and interventions.

The aim of this study was to analyse health promoting lifestyle in conjunction with socio-demographic conditions and Facebook usage and describe the complex links between these variables using non-linear canonical correlation analysis.

## Materials and Methods

### Participants and procedure

We used a cross-sectional, correlational study design. This study regarded Facebook as a social media tool. The population of the study was made up of Facebook users in Ankara, Turkey. With this goal, using the advanced search method on Facebook, a list of the users living in Ankara was compiled, and a questionnaire was sent to 4014 randomly selected users between 10 January to 10 April 2018, and 506 replies were received. From the 506 users, after removing the users who did not accept the study’s friendship requests or who replied to the survey with missing data, 423 Facebook users were included in the survey. Based on the results of his study, Thompson^17^ suggests that researchers should attempt to employ at least 10 subjects per variable in multivariate studies. He also reported that canonical results are not positively biased, especially if sample size is at least 10 subjects per variable. So, the sample size with 160 (16*10) and more participants were considered sufficient.

### Measures

The collection of data was carried out in two stages. In the first stage, a questionnaire, including the Health-Promoting Lifestyle Profile II (HPLP II), developed by Pender, and Turkish reliability and validity studies conducted by Bahar et al,^18^ were prepared to obtain data on participants’ socio-demographic characteristics and healthy lifestyle behaviours. HPLP II is a four-point Likert-scale behaviour questionnaire with 52 items that measures the frequency of self-reported health promoting behaviours. The six behavioural dimensions consist of self-actualization, health responsibility, exercise, nutrition, interpersonal support and stress management. The created questionnaire form was provided to be filled out by Facebook users through an online link sent to them via message. In the second stage, data were collected to identify participants’ Facebook usage indicators, which were defined as: *friends:* the number of Facebook friends, *posts* : participants’ number of posts in one month, *like rate and comment rate* : total number of likes and comments on participants’ posts in one month, *pages* : the number of pages users followed through likes, and *groups* : the number of groups in which they have participated. Since the process of obtaining related data required a lengthy and laborious effort, each participant was not directly questioned; instead, the information was personally determined by the researchers. In order to reach the data, friendship requests were sent to the users who completed the questionnaire within the scope of the research, and the ones who accepted the request were included in the study.

OVERALS allows us to include different sets in the analysis. In this analysis, we used three sets: set one includes socio-demographic variables, set two consists of social media usage indicators, and set three includes the six sub-dimensions of the HPLP II. The socio-demographic variables included gender, age, education and marital status. Age was categorized into three groups: 18-34, 35-64, 65 and above. Education was categorized as less than high school degree, high school degree and more than high school degree. Marital status was categorized as single and married.

### Statistical analysis

The statistical analyses were performed using SPSS 22.0 for Windows (SPSS, Inc., Chicago, IL, USA). At ﬁrst, descriptive statistics were calculated, then HPLP sub-dimensions and Facebook usage indicators were categorized for use in nonlinear canonical correlation analysis (OVERALS). Those who are below the median score in the HPLP sub-dimensions (these are health responsibility, self-actualization, nutrition, exercise, interpersonal support, and stress management) were grouped as “negative”, while the ones with median and higher values were grouped as “positive”. The Facebook usage indicators (these are the number of friends, posts, page likes, groups, like rate, and comment rate for one month) below the median value were classified as “low”, while those with equal or higher than the median value were classified as “high”. The differences in demographic characteristics and categorized health and social factors were evaluated with non-parametric Pearson chi-square or maximum likelihood ratio chi-square tests.

OVERALS analysis was used to explore relationships between health behaviours, social media usage and demographic characteristics. Classical canonical correlation analysis is utilized when the variables are measured numerically or when the multivariate normal distribution assumption is met. However, OVERALS is proposed for non-numerical variable sets in scale studies such as those found in health or social sciences where these conditions are not met. This procedure allows for the graphical representation of relationships among a large number of variables belonging to more than two sets. The purpose is to detect how similar sets of variables are to each other. The great advantage of OVERALS compared to other multivariate techniques is that variables with different scaling levels such as nominal, ordinal, and numerical levels can be included in an analysis of nonlinear relationships between variables. ^19,20^

The interpretation of the results contains evaluation of the fit and the loss of the solution, the eigenvalues, the weights, and the component loadings, as well as the centroid plot presentation providing the assessment of associations among the categories.^19,21^

## Results

This study included 423 participants and 51.1% of them were female, 48.9% were male. Single participants made up 53.9%, while 46.1% were married. In this study sample, 26.5% respondents were at less than high school level, 59.6% were at high school level and 13.9% were at more than high school level. 30.5% of all participants were in the age group of 18-34, 49% were in the age group of 35-64 and 20.5% were 65 of age or older.

Associations between health promoting lifestyles and sociodemographic factors are shown in Table 1. Health promoting lifestyles by gender were similar. There were no significant differences of health promoting lifestyle subscales in terms of gender categories.

We did not find significant differences at health promoting lifestyle scores in terms of age groups except for exercise subscale (*P* = 0.018). Younger groups engaged more in physical activity behaviors than did older group.

There were no significant differences of health promotion subscales according to level of education except for self-actualization subscale (*P* = 0.014). Respondents who had more education (more than high school degree) showed more positive self-actualization behaviour than those who had less education (high school degree or less).

There were no statistically significant differences in self-actualization, nutrition, interpersonal support, and stress management subscales according to marital status. Significant differences were revealed in the health responsibility (*P* = 0.004) and physical activity (*P* = 0.027) subscales. Singles engaged more in physical activity and health responsibility (39.5% and 41.2%, respectively) behaviors than did married persons (29.2% and 27.7%, respectively).

Table 2 represents associations between Facebook usage variables and sociodemographic factors. We found significant differences for number of Facebook friends, posts, and groups scores according to gender categories. There were significant differences for Facebook posts, groups, like rate, and comment rate scores in terms of age categories. There were also significant differences for some Facebook variables: friends, posts and like rate according to education levels. There were significant differences for Facebook posts and comment rate in terms of marital status.

The number of friends was higher in males and increases in proportion to education level. *Posts* were at the lowest level in young people (28.7%) and increased in proportion to age (52.7% and 72.4, respectively). As the education level increased, the like rate also increased. *Posts* were at a higher level in married people (56.9%) than singles (43%). *Page likes* were higher for males (55.6%) than females (44%). *Groups* were higher for males (50.2%) and significantly increased with age (33.3%, 48.3%, and 55.2, respectively). The *like rate* of users decreased with age (58.1%, 49.3%, and 39.1, respectively). Similarly, a high level of *like rate* increased dependent on their level of education (Low: 40.2%, graduated: 51.2%, post graduate: 62.7%; respectively). Middle-aged people had more *comments* (55.6%) than young and older people. The high rate of *comments* received by married couples (52.3%) was higher than that of singles.

Since all the data are non-numerical, OVERALS was applied to identify the relationships between three sets of variables, and a two-dimensional solution was found. Component loadings, loss values, eigenvalues and fit values that show the similarities between the sets obtained from the OVERALS are given in Table 3.

Of the relationship captured between the variables, 0.487/0.958 = 51% was explained by the first dimension and 0.472/0.958 = 49% was explained by the second. The canonical correlation coefficients which are the square roots of these eigenvalues were 0.70 and 0.69 for first and second dimensions, respectively. These values represented a positive and high relationship between the variable clusters discussed in both dimensions. The total fit measure was equal to 0.958 and explained 0.958/2 = 48% of the total variance when compared to the maximum fit (2), which is equal to the number of dimensions.

**Figure 1:**
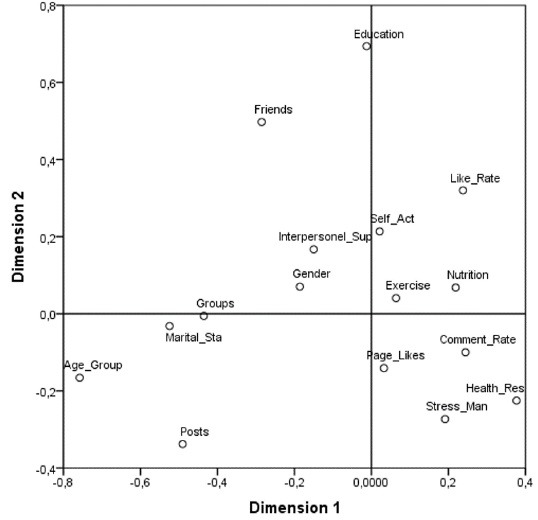
Component loadings of OVERALS analysis.

When looking at the graph of component loadings (Figure 1), it can be seen that the best discriminatory power belongs to education level, age group, number of friends, like rate, health responsibility and stress management variables, because these variables were located away from the origin. From the component loadings of Table 3; health responsibility, stress management, page likes, nutrition, and age group took high values in the first component. Page likes, like rate, friends, education, interpersonal support, and self-actualization had high component values in the second dimension.

Table 3 Component loadings, eigenvalues among the demographic, Facebook usage indicators and health promoting lifestyle.

**Figure 2:**
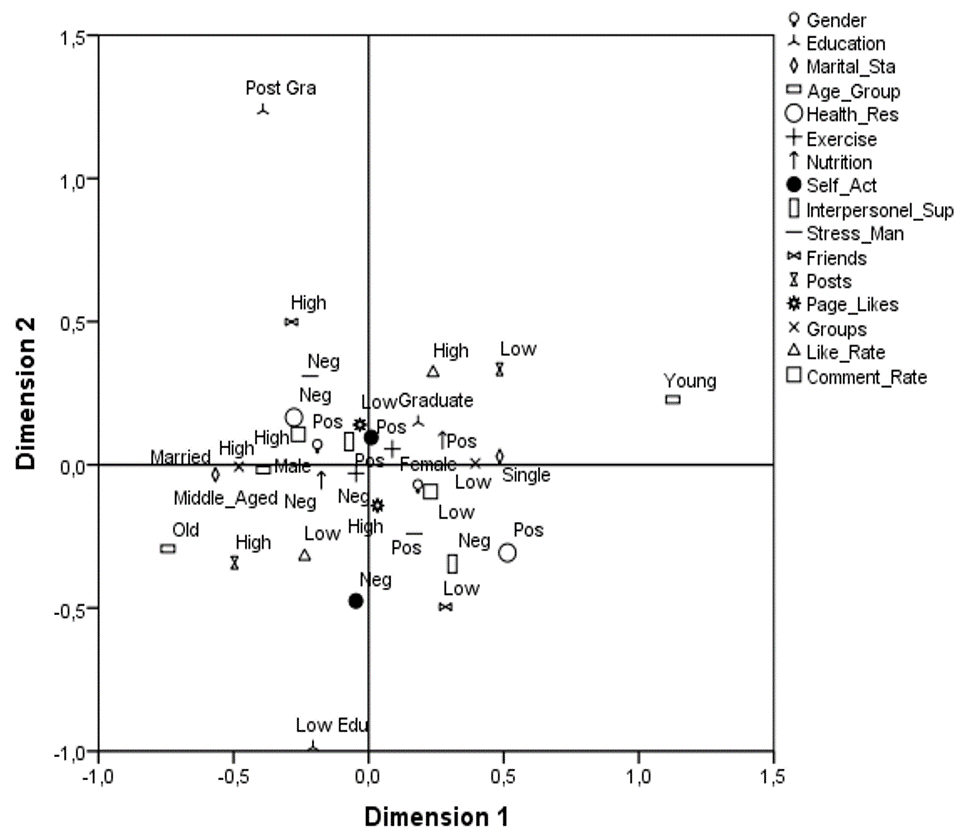
Centroids plot for the OVERALS analysis of the relationship between basic demographics, healthy lifestyle factors and social media tools.

A detailed demonstration of the compatibility among the categories is given in Figure 2. These point distributions provide us with the ability to identify key relationships between demographic variables, Facebook usage indicators and health-promoting lifestyle sub-dimensions.

According to both the component loadings and centroid plot, there was a positive relationship between self-actualization, exercise, like rate, and nutrition. In the first coordinate, while the self-actualization, nutrition and exercise were positive, the like rate is high and the posts tended to be low. In contrast, in the third coordinate, it was noticed that those who had negative self-actualization, exercise and nutrition statuses had high levels of posts and low levels of like rates. This group mostly contained old men. In the second coordinate, it is seen that those whose number of friends and comment rate were high and who have had positive interpersonal supports had negative levels of health responsibility and stress management. It can be said that the self-actualization of this group, which was located in the second coordinate, was positive, and the level of education was concentrated in the graduated or post graduate level. In contrast, when the fourth coordinate is examined; those with a low level of friends, low like rates, and little group participation tended to be positive in stress management and health responsibility, although the interpersonal support was negative. Posts, groups and comment rate of young and single people were low, but their like rate was very high. On the other hand, married people had very high levels of posts and groups, whereas their like rate was low. While the self-actualization and interpersonal support of people with lower education levels were inclined to be negative, the number of friends was also low. However, as it is seen from the third coordinate, this group had a high number of posts. It was noteworthy that the people with the less than high school level had a higher number of friends, and their self-actualization and interpersonal support were more positive. At coordinates 2 and 3, the group of mostly middle-aged men with a high rate of group participation and high comment rate was found to be likely to be negative about exercise, nutrition and even health responsibility. On the first and fourth coordinates, the opposite of the previous ones, the group consisted mostly of single women with low group participation and low comment rate who tended to be positive in stress management, nutrition and exercise.

## Discussion

This study is one of the first to emerge regarding the relationships between Facebook usage and a health promotion lifestyle, including health responsibility, nutrition, exercise, self-actualization, interpersonal support, and stress management. A strong aspect of the study is the use of the OVERALS analysis, which makes it possible to formally assess the relationship between people’s demographic characteristics, healthy lifestyle behaviours and social media use.

A monthly observation based on users’ sharing posts and receiving likes or comments on their posts shows that those who are positive in self-actualization, nutrition and exercise have a high level of like rate, while their number of posts is low. On the contrary, it is noted that those who have negative self-actualization, exercise and nutrition status have a high number of posts and a low level of like rates. In addition, females have better behaviours with respect to exercise and nutrition than males, and they get more likes on their posts even though they share fewer posts. This can be explained by the phenomenon of physical attractiveness. Because two important cues for female and male physical attractiveness are body mass index and shape,^22,23^ and those people of higher physical attractiveness are perceived to possess more socially desirable traits.^24^ On the other hand, those with high education levels are more positive in exercise, self-actualization and interpersonal support, while negative in health responsibility and stress management.

Some additional evidence was provided by our study on Facebook friend numbers and social support patterns. People with more friends on Facebook have more positive interpersonal support and self-actualization. This finding is similar to studies that reported that the number of Facebook friends emerged as a stronger predictor of perceived social support and may enhance a user’s subjective well-being.^16,25,26^ Overall, independent from the number of Facebook posts, people who get more comments and likes on their posts and have more Facebook friends are worse off in stress management and health responsibility. This finding is in line with the study reported by Chen and Lee,^27^ which showed that more frequent Facebook interaction (including sharing photos and new stories, liking and commenting) is associated with greater distress. Therewithal, Campisi et al^28^ suggested an association between Facebook use and psychological stress.

There were some limitations in the current study. This study assessed only Facebook users living in Ankara region. Our results could not be generalized to other social media tools users and other parts of the country. Replicated studies with other social media tools user groups residing in different geographical regions are needed to verify and compare the present results. Additionally, this study has been conducted with a sample from Ankara in Turkey, which is a part of a collective society. We also need more studies to be carried out in different cultural backgrounds in order to consider the results complete. Due to increasing time spent on social media in all societies, it is important for public health researchers and health policy makers to understand the relationship between social media usage and health behaviours.

In conclusion, our results highlight data showing that older people share significantly more posts than young people and have lower like rates. Additionally they have negative nutrition and exercise behaviors. We think that it is a result of social isolation, which is a common factor among the elderly population. Based on study findings it seems that there is a problem in health promoting behaviors of the older Facebook users. We recommend creating environments for older adults that are conductive to more social lifestyle. Additionally, at each clinic visit, the health provider should make suggestions for increasing physical activity. Also, educational programs encouraging healthy eating should be developed.

## Ethical approval

This study was approved by Hacettepe University Ethics Commission (grant No. 35853172-900 ) and informed consent obtained from all participants.

## Competing interests

The authors declare that they have no competing interests.

## Funding

No funding was provided for this research.

## Authors’ contributions

DG developed the framework, concept and design of the research project, collected the data. SYI performed analysis and had contribution in data entering phase. DG and SYI interpreted the data, wrote the results and revised the manuscript. DG approved the final version of the manuscript. EO has full supervision on the whole research project. All authors discussed and contributed to the final manuscript.

## Acknowledgments

The authors greatly appreciate all the people who participated in the study for their support. This article was provided from PhD dissertation of Güleç.

**Table 1:** Distribution of health promoting lifestyle subscales by sociodemographic groups

		**Self-Actualization**		**Health Responsibility**		**Nutrition**		**Exercise**		**Interpersonal Support**		**Stress Management**	
		**Neg.** **No. (%)**	**Pos.** **No. (%).**	**Neg.** **No. (%)**	**Pos.** **No. (%).**	**Neg.** **No. (%)**	**Pos.** **No. (%).**	**Neg.** **No. (%)**	**Pos.** **N( %)**	**Neg.** **No. (%)**	**Pos.** **No. (%).**	**Neg.** **No. (%)**	**Pos.** **No. (%).**
**Gender**	Female 216 (51.1%)	37 (17.1)	179 (82.9)	139 (64.4)	77 (35.6)	129 (59.7)	87 (40.3)	142 (65.7)	74 (34.3)	43 (19.9)	173 (80.1)	101 46.8	115 (53.2)
	Male 207 (48.9%)	34 (16.4)	173 (83.6)	136 (65.7)	71 (34.3)	129 (62.3)	78 (37.7)	134 (64.7)	73 (35.3)	37 (17.9)	170 (82.1)	84 (40.6)	123 (59.4)
	*P* ^a^	0.846		0.838		0.584		0.828		0.594		0.200	
**Age (y)**	18-34 129 (30.5%)	18 (14)	111 (86)	76 (58.9)	53 (41.1)	71 (55)	58 (45)	80 (62)	49 (38)	23 (17.8)	106 (82.2)	50 (38.8)	79 (61.2)
	35-64 207 (49%)	40 (19.3)	167 (80.7)	137 (66.2)	70 (33.8)	127 (61.4)	80 (38.6)	128 (61.8)	79 (38.2)	42 (20.3)	165 (79.7)	89 (43)	118 (57)
	65+ 87 (20.5%)	13 (14.9)	74 (85.1)	62 (71.3)	25 (28.7)	60 (69.0)	27 (31)	68 (78.2)	19 (21.8)	15 (17.2)	72 (82.8)	46 (52.9)	41 (47.1)
	*P* ^a^	0.384		0.155		0.119		0.018*		0.773		0.117	
**Education**	Less than high school level 112 (26,5%)	22 (19.6)	90 (80.4)	67 (59.8)	45 (40.2)	67 (59.8)	45 (40.2)	78 (69.6)	34 (30.4)	21 (18.8)	91 (81.3)	47 (42)	65 (58)
	High school level 252 (59,6%)	46 (18.3)	206 (81.7)	166 (65.9)	86 (34.1)	153 (60.7)	99 (39.3)	161 (63.9)	91 (36.1)	50 (19.8)	202 (80.2)	108 (42.9)	144 (57.1)
	More than high school level 59 (13,9)	3 (5.1)	56 (94.9)	42 (71.2)	17 (28.8)	38 (64.4)	21 (35.6)	37 (62.7)	22 (37.3)	9 (15.3)	50 (84.7)	30 (50.8)	29 (49.2)
	*P* ^a^	0.014*		0.301		0.833		0.511		0.710		0.488	
**Marital Status**	Single 228 (53,9%)	38 (16.7)	190 (83.3)	134 (58.8)	94 (41.2)	136 (59.6)	92 (40.4)	138 (60.5)	90 (39.5)	48 (21.1)	180 (78.9)	91 (39.9)	137 (60.1)
	Married 195 (46,1%)	33 (16.9)	162 (83.1)	141 (72.3)	54 (27.7)	122 (62.6)	73 (37.4)	138 (70.8)	57 (29.2)	32 (16.4)	163 (83.6)	94 (48.2)	101 (51.8)
	*P* ^a^	0.944		0.004**		0.540		0.027*		0.224		0.087	

^a^ Chi-Square; * * and * indicate significance at 0.01 and 0.05, respectively.

**Table 2:** Distribution of Facebook usage variables by sociodemographic groups

		**Friends**		**Posts**		**Pages**		**Groups**		**Like Rate**		**Comment Rate**	
		**Low** **No. (%)**	**High** **No. (%)**	**Low** **No. (%)**	**High** **No. (%)**	**Low** **No. (%)**	**High** **No. (%)**	**Low** **No. (%)**	**High** **No. (%)**	**Low** **No. (%)**	**High** **No. (%)**	**Low** **No. (%)**	**High** **No. (%)**
Gender	Female 216 (51.1%)	128 (59.3)	88 (40.7)	115 (53.2)	99 (47.8)	121 (56)	95 (44)	129 (59.7)	87 (40.3)	103 (47.7)	113 (52.3)	118 (54.6)	98 (45.4)
	Male 207 (48.9%)	84 (40.6)	123 (59.4)	101 (46.8)	108 (52.2)	92 (44.4)	115 (55.6)	103 (49.8)	104 (50.2)	103 (52.7)	98 (47.3)	107 (51.7)	100 (48.3)
	*P* ^a^	0.001**		0.266		0.017*		0.040*		0.307		0.545	
Age(y)	18-34 129 (30.5%)	74 (57.4)	55 (42.6)	92 (71.3)	37 (28.7)	64 (49.6)	65 (50.4)	86 (66.7)	43 (33.3)	54 (41.9)	75 (58.1)	85 (65.9)	44 (34.1)
	35-64 207 (49%)	101 48.8	106 51.2	98 (47.3)	109 (52.7)	111 (53.6)	96 (46.4)	107 (51.7)	100 (48.3)	105 (50.7)	102 (49.3)	92 (44.4)	115 (55.6)
	65+ 87 (20.5%)	37 (42.5)	50 (57.5)	24 (27.6)	63 (72.4)	38 (43.7)	49 (56.3)	39 (44.8)	48 (55.2)	53 (60.9)	34 (39.1)	48 (55.2)	39 (44.8)
	*P* ^a^	0.088		0.001**		0.292		0.003**		0.022*		0.001	
Education	Less than high school level 112 (26,5%)	72 (64.3)	40 (35.7)	43 (38.4)	69 (61.6)	54 (48.2)	58 (51.8)	63 (56.3)	49 (43.8)	67 (59.8)	45 (40.2)	58 (51.8)	54 (48.2)
	High school level 252 (59,6%)	119 (47.2)	133 (52.8)	139 (55.2)	113 (44.8)	123 (48.8)	129 (51.2)	130 (51.6)	122 (48.4)	123 (48.8)	129 (51.2)	143 (56.7)	109 (43.3)
	More than high school level 59 (13,9)	21 (35.6)	38 (64.4)	32 (54.2)	27 (45.8)	36 (61)	23 (39)	39 (66.1)	20 (33.9)	22 (37.3)	37 (62.7)	24 (40.7)	35 (59.3)
	*P* ^a^	0.001**		0.011*		0.209		0.123		0.016*		0.079	
Marital status	Single 228 (53,9%)	109 (47.8)	119 (52.2)	130 (57)	98 (43)	108 (47.4)	120 (52.6)	132 (57.9)	96 (42.1)	108 (47.4)	120 (52.6)	132 (57.9)	96 (42.1)
	Married 195 (46,1%)	103 (52.8)	92 (47.2)	84 (43.1)	111 (56.9)	105 (53.8)	90 (46.2)	100 (51.3)	95 (48.7)	104 (53.3)	91 (46.7)	93 (47.7)	102 (52.3)
	*P*	0.304		0.004**		0.184		0.173		0.221		0.036	

^a^ Chi-Square; * * and * indicate significance at 0.01 and 0.05, respectively.

**Table 3:** Component loadings, eigenvalues among the demographic, Facebook usage indicators and health promoting lifestyle

**Set**	**Variable**	**Dimension**		
		**1**	**2**	
1	Gender^a,b^	-0.186**	0.070	
	Education^b,c^	-0.013	0.694**	
	Marital status^a,b^	-0.524**	-0.032	
	Age group^b,c^	-0.758**	-0.166**	
2	Health responsibility^a,b^	0.377**	-0.225**	
	Exercise^a,b^	0.064	0.041	
	Nutrition^a,b^	0.219**	0.068	
	Self actualization^a,b^	0.021	0.214**	
	Interpersonal support^a,b^	-0.150**	0.167**	
	Stress management^a,b^	0.191**	-0.273**	
3	Friends^a,b^	-0.285**	0.497**	
	Posts^a,b^	-0.491**	-0.338**	
	Pages^a,b^	0.032	-0.141**	
	Groups^a,b^	-0.435**	-0.006	
	Like rate^a,b^	0.238**	0.320**	
	Comment rate^a,b^	0.244	-0.100*	
	Sets	1	2	Total
Loss	Set 1	0.397	0.445	0.842
	Set 2	0.786	0.699	1.485
	Set 3	0.358	0.440	0.798
	Mean	0.513	0.528	1.042
Eigenvalue		0.487	0.472	
Fit			0.958

* 0.05, ** 0.01 level of significance of the point-biserial correlations. ^a^ Optimal scaling level: single nominal. ^b^ Projections of the single quantiﬁed variables in the object space. ^c^ Optimal scaling level: ordinal.
